# Postoperative radiotherapy versus postoperative radiochemotherapy after surgery of salivary gland cancer: a systematic review and meta-analysis

**DOI:** 10.1038/s41598-026-52018-4

**Published:** 2026-05-06

**Authors:** Anja Wilhelmy, Peter Schlattmann, Orlando Guntinas-Lichius

**Affiliations:** 1 Department of Otorhinolaryngology, Jena University Hospital, Am Klinikum 1, 07747 Jena, Germany; 2 Department of Medical Statistics, Computer Sciences and Data Sciences, Jena University Hospital, 07747 Jena, Germany; 3 Facial-Nerve-Center, Jena University Hospital, 07747 Jena, Germany

**Keywords:** Salivary glands, Parotid gland cancer, Submandibular gland cancer, Overall survival, Adjuvant therapy, Chemotherapy, Toxicity, Cancer, Medical research, Oncology

## Abstract

There is a lack of prospective clinical trials on the therapy of salivary gland cancer (SGC) due the rarity and large variety of histological subtypes. This study aimed to compare overall survival (OS) after primary surgery of SGC followed by postoperative radiotherapy (PORT) versus radiochemotherapy (PORCT) in curative intent. A systematic review and meta-analysis were performed on studies reporting OS after the two postoperative therapy modalities using hazard ratios (HRs) or allowing to calculate the HRs from the data. These studies were identified from PubMed, Web of Science, and Cochrane Library databases until October 2024. Pooled HR with 95% confidence interval (CI) is reported with random-effects or fixed-effects models. The search yielded 1,074 publications, of which 11 retrospective clinical studies with 26,612 adult patients could be included. None of the studies found a statistically significant difference between PORT and PORCT. Likewise, this meta-analysis revealed no statistically significant difference between PORT and PORCT. The pooled HR based on a common effect model was 1.065 (CI: 0.998–1.137) with low between study heterogeneity (I²=24.1%) and heterogeneity variance τ^2^ = 0.009. A meta-regression only revealed one factor with influence on the HR. The proportion of T4 patients in the total study shows a HR = 1.153 for a 10% change 95% CI: 1.014,1.314). This meta-analysis, based on a highly confounded observational setting, does not permit a reliable conclusion as to whether PORCT leads to better OS than PORT as adjuvant therapy for patients with SGC. PORCT might be beneficial for high-risk subgroups. However, this was not possible to determine in this meta-analysis as all included studies were retrospective and heterogeneous leading to a high degree of uncertainty about the therapy effects. More robust data from high-quality cohort studies or randomized controlled studies are needed. There is an urgent need for alternative and more effective multimodal treatment concepts for SGC.

**Supplementary Information:**

The online version contains supplementary material available at 10.1038/s41598-026-52018-4.

## Introduction

Surgery is the standard primary curative treatment for patients with salivary gland cancer. Postoperative radiotherapy is administered to patients with tumor characteristics such as specific histological subtypes, advanced stage, and high-risk pathological factors^1–3^. Based on the results of randomized controlled phase III trials, high-risk criteria have been defined for the use of postoperative radiochemotherapy instead of radiotherapy for the most common types of head and neck squamous cell cancer, i.e., cancer of the oral cavity, oropharynx, hypopharynx and larynx, in order to reduce the risk of recurrence and prolong overall survival^4^. Due to diverse and highly variable histologies of salivary gland cancer, this much rarer type of head and neck cancer is typically excluded from prospective randomized clinical trials. Nevertheless, as the 5-year overall survival of salivary gland cancer is, on average, less than 50%^5^, and given the risk of late distant metastasis, there is a need to improve adjuvant treatment – for example, by using postoperative radiochemotherapy also in high-risk subsets of salivary gland cancer. To date, the effect of postoperative radiochemotherapy compared with postoperative radiotherapy has only been analyzed in retrospective observational trials, yielding variable and contradictory results.

Therefore, the primary aim of the present study was to perform a systematic review and meta-analysis of overall survival following surgery for salivary gland cancer, comparing postoperative radiotherapy (PORT) with postoperative radiochemotherapy (PORCT). The secondary aim was to conduct a systematic review of the toxicity associated with postoperative radiotherapy versus postoperative radiochemotherapy. Due to the frequently missing or incomplete information on treatment-related side effects, as well as the use of different classification systems used by the respective study authors, a meta-analysis for the secondary aim was not feasible.

## Materials and methods

This study followed the Preferred Reporting Items for Systematic Reviews and Meta-Analyses (PRISMA) guidelines^6^. The PRISMA checklist can be found in Supplementary Material 1. Ethical approval and patient informed consent were not required for a meta-analysis.

### Inclusion and exclusion criteria

Where available, randomized controlled trials and cohort studies were included. Only studies on primary salivary gland malignancies were considered. Patients with disease recurrence and distant metastases at the time of diagnosis were excluded. Patients had to be at least 18 years old, as the indication for postoperative therapy is more critical in pediatric cases due to secondary damage^7^. Only studies in which primary tumor resection was followed by postoperative radiotherapy (PORT) or radiochemotherapy (PORCT) were included. Studies involving neoadjuvant therapies or non-surgical treatment were excluded. If several studies used data from the same database, they were still included as long as they differed from each other in their inclusion criteria.

### Endpoints

The primary endpoint of the study was the comparison of overall survival between the two postoperative therapy modalities expressed as a hazard ratio (HR). The secondary endpoint was treatment toxicity. The original aim was to investigate toxicity quantified according to the Common Terminology Criteria for Adverse Events (CTCAE)^8^, also by means of a meta-analysis. Due to the frequently missing or incomplete information on treatment-related side effects, as well as the use of different classification systems by the authors, only a systematic review was conducted for the secondary endpoint instead.

### Data sources and literature search

The databases PubMed (https://pubmed.ncbi.nlm.nih.gov/), Web of Science (https://www.webofscience.com/wos/), and the Cochrane Library (https://www.cochranelibrary.com/) were searched using a search strategy defined in advance. In addition, a manual search was conducted in the references and citing publications of the included studies, as well as in related review articles and study registries, to identify grey literature. Grey literature refers to unpublished works, such as conference abstracts, results from ongoing studies, or dissertations. The literature search was last updated on October 8, 2024. The following Medical Subject Heading (MeSH) terms and combinations were used: (a) in PubMed: (salivary gland tumor[MeSH Terms] OR salivary gland cancer[MeSH Terms] OR salivary gland neoplasm[MeSH Terms] OR parotid cancer[MeSH Terms] OR submandibular cancer[MeSH Terms] OR neoplasm, sublingual gland[MeSH Terms]) AND (adjuvant radiotherapy[MeSH Terms] OR adjuvant radiochemotherapy[MeSH Terms] OR adjuvant chemoradiotherapy[MeSH Terms] OR radiotherapy, adjuvant/adverse effects[MeSH Terms] OR chemoradiotherapy, adjuvant/adverse effects[MeSH Terms] OR radiochemotherapy, adjuvant/adverse effects[MeSH Terms]); (b) in the Web of Science: (ALL=(salivary gland tumor) OR ALL=(salivary gland cancer) OR ALL=(salivary gland neoplasm) OR ALL=(parotid cancer) OR ALL=(submandibular cancer) OR ALL=(neoplasm, sublingual gland)) AND (ALL=(adjuvant radiotherapy) OR ALL=(adjuvant radiochemotherapy) OR ALL=(adjuvant chemoradiotherapy) OR ALL=(adjuvant radiotherapy, adverse effects) OR ALL=(adjuvant radiochemotherapy, adverse effects) OR ALL=(adjuvant chemoradiotherapy, adverse effects)), and (c) in the Cochrane Library: (salivary gland tumor OR salivary gland cancer OR salivary gland neoplasm OR parotid cancer OR submandibular cancer OR neoplasm, sublingual gland) AND (adjuvant radiotherapy OR adjuvant radiochemotherapy OR adjuvant chemoradiotherapy OR adjuvant radiotherapy, adverse effects OR adjuvant chemoradiotherapy, adverse effects OR adjuvant radiochemotherapy, adverse effects). The literature search revealed 1074 results until 08-October 2024.

### Selection of studies

Two independent reviewers (A.W.; O.G.L.) reviewed abstracts and full texts. In cases of disagreement, a joint decision was made in a discussion. All studies were assessed against general exclusion criteria: review articles, duplicate patients, absence of essential data, multiple use of same patient dataset, and animal studies. Further exclusion criteria were as follows: non-English or non-German language; full text not available; insufficiently reported data or non-extractable data; case series including fewer than ten patients; subgroup analyses of patients from larger studies; article types including reviews, case reports, conference abstracts, letters to the editor, or book chapters. No restrictions on the publication date were applied, but peer-reviewed journal publication was a requirement for article inclusion.

### Eligibility criteria

**Fig. 1:**
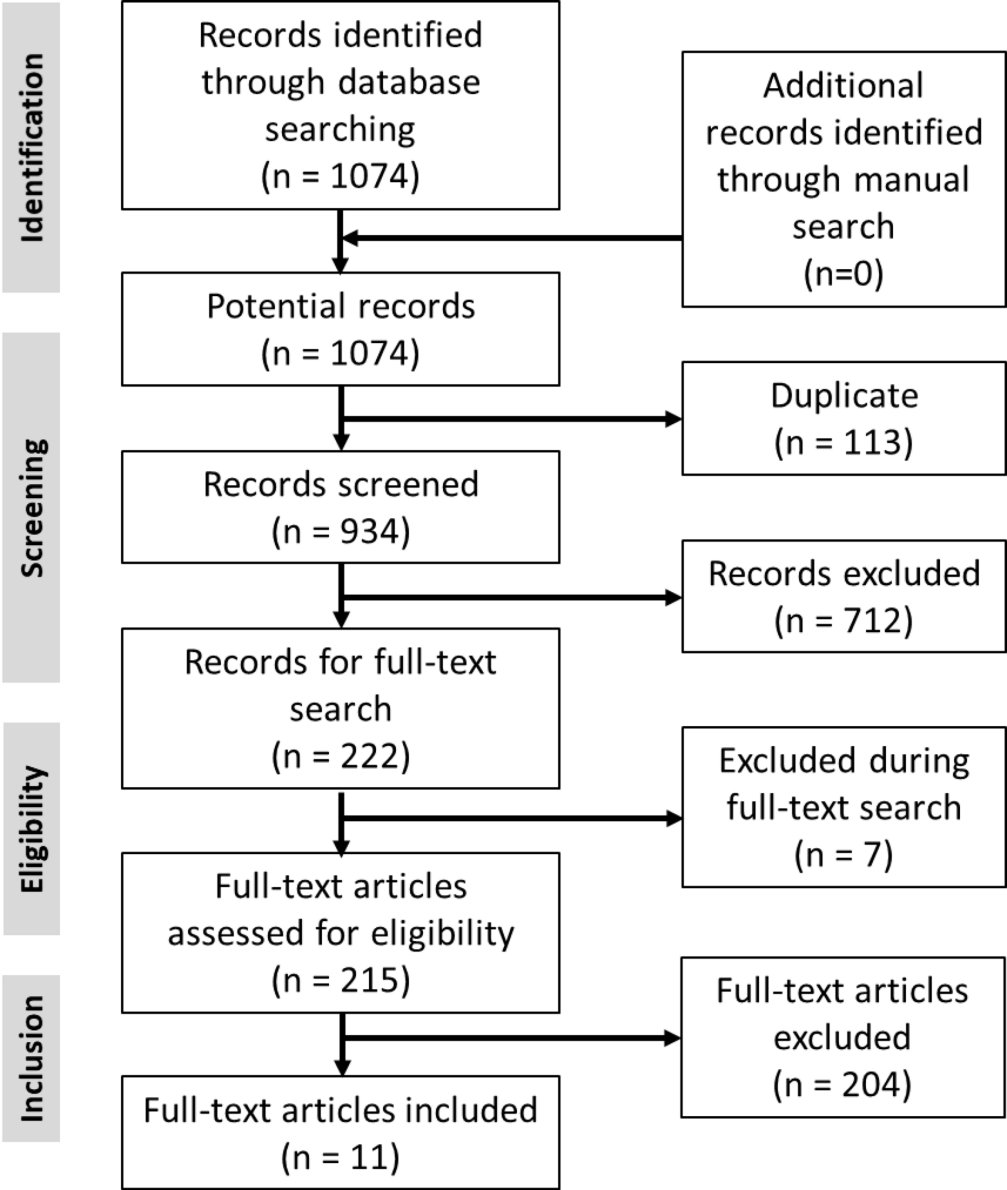
PRISMA chart showing the process of the selection of the included studies.

The PICOS scheme was utilized to establish the eligibility criteria for this study. Patients (P): ≥18 years of age, with malignant primary salivary gland cancer. Intervention (I): primary surgery with curative intent followed by postoperative radiotherapy (PORT). Comparison (C): primary surgery with curative intent followed by postoperative radiochemotherapy (PORCT). Outcomes (O): Two outcomes were analyzed: overall survival and treatment toxicity. To compare the overall survival for the two treatment concepts, each study was checked to determine whether a hazard ratio (HR) was given or could be calculated from the available data^9^. Study design (S): Retrospective and prospective cohort studies, cohort studies, case series, and randomized clinical trials (RCTs) were included. Eleven (11) articles were finally included in the analysis (Fig. 1). The reasons for excluding full-text studies are summarized in Supplementary Table 1.

### Data extraction and quality analysis

The HRs with confidence intervals (CI) and p-values (p) as well as the sample sizes for the treatment modalities were summarized for each of the studies. The HR indicates the ratio of the respective hazards of the two treatment groups^10,11^. In addition, the key figures of the patient cohorts on tumor and lymph node classification according to the TNM classification, histology, chemotherapeutic agent used, radiation technique and dose as well as the publication year of the study were documented. To improve comparability between the studies, information on tumor and lymph node classification was standardized to percentages of the total cohorts and the postoperative therapy groups.

The Newcastle–Ottawa Quality Assessment Scale (NOQAS) for cohort studies (http://www.ohri.ca/programs/clinical_epidemiology/oxford.asp) was used by two reviewers (A.W. and O.G.L.) to assess the overall quality of each included study. The two reviewers evaluated the implementation of this assessment tool and agreed on a method before their independent assessments of the studies. Disagreements were resolved by discussion. The results are presented in Supplementary Table 2. The NOQAS score was high for 6 studies and moderate for 5 studies.

### Statistics

Statistical analysis was performed in RStudio, Version 4.3.; www.r-project.org). The package meta^12^, metafor^13^, dmetar^14^ and metasens^15^ were used for statistical calculations. Not all studies were considered to the same extent. The weighting with which the individual studies were included in the pooled effect was determined via the inverse variance. Thus, publications with larger samples and a more precise estimate of the observed effect had a greater influence on the pooled effect of the meta-analysis than small studies with a high standard deviation. The proportion of favorable results is presented in the forest plots as hazard ratio (HR) together with a 95% confidence interval (CI). We aimed to use a parsimonious model based on statistical grounds. First, we calculated a common effect and a model with random effects using the restricted maximum likelihood method in order the estimate the heterogeneity variance. In order to compare the fit of the respective models we applied the likelihood ratio test. This resulted in a value of 1.2613 with df = 1 and *p* = 0.261. Thus, the common effect model turned out to be the most parsimonious model. For that matter we decided to report common effect model as primary analysis^16^. Importantly, the random-effects model yielded virtually identical results (HR 1.087, 95% CI 0.985–1.200), supporting the robustness of the findings regardless of the modeling approach chosen; both models are reported to allow clinical interpretation in the context of between-study variability. Assessment of statistical heterogeneity was performed using Cochran’s Q-test with corresponding I² statistic^17^. This statistic is given as a percentage, and represents the variability due to heterogeneity between studies. As a sensitivity analysis a random-effects model was applied where the heterogeneity variance (τ²) was estimated via restricted maximum likelihood (REML). Publication bias was assessed via Egger’s test for funnel plot asymmetry^18^. In the leave-one-out analysis, the result of the meta-analysis was recalculated, in each case excluding one of the studies.

## Results

### Included studies

The eleven included studies are listed in Table 1. These studies included 26,612 patients and are all retrospective cohort studies. No randomized controlled trial could be found. None of these eleven studies found a significant difference between PORT and PORCT. Five of the studies obtained their patient data from National Cancer Database (NCDB) but used different time periods^21–25^. Six studies included only cancer of the major salivary glands. Three studies included major and minor salivary gland cancer. One trial was focused on minor salivary gland cancer and one on sublingual gland cancer. The majority, i.e. eight studies included all histologies. Two studies were focused on adenoid cystic carcinoma and another on salivary duct cancer. In four studies, the T classification was specified separately for both intervention groups. In six the N classification was mentioned. The proportions of T4 tumors and patients with lymph node metastases (N+) were higher in the PORCT group than in the PORT group.

**Table 1:** Included studies. All studies were retrospective cohort studies. No randomized controlled trials could be identified.

Study	Year	Localization	Histology	Ratio T(3/4) %	Ratio T4 PORCT vs. PORT	Median FU	Ratio *N*+ PORCT vs. PORT	RT (*n*)	RCT (*n*)	HR (RCT/RT)	95% CI	*p*
Amini et al^21^.	2016	Major SGC	all	60.9	1.45	3.3 years	1.59	1842	368	1.22	1.03–1.44	0.02
Cheraghlou et al^22^.	2018	Major SGC	all	39.2	NA	NA	NA	193	834	1.023	NA	0.725
Gordon et al^23^.	2023	Major SGC	all	37.3	2.48	5.1 years	6.02	13,601	2355	0.923	0.784–1.087	0.34
Kang et al^44^.	2022	Major SGC	all	82.8	1.91	3.4 years	2.02	217	178	0.94	0.72–1.23	0.6654
Onderdonk et al^27^.	2020	Major and minor SGC	all	46.0	5.33	4.3 years	5.8	50	58	1.529	NA	0.42
Qiu et al^28^.	2024	Sublingual SGC	ACC	62.1	NA	6.3 years	1.06	147	59	1.27	0.636–2.573	0.49
Tanvetyanon et al^29^.	2016	Major and minor SGC	all	NA	NA	3.3 years	1.14	641	100	1.39	1.07–1.79	0.12
Torabi et al^24^.	2020	Minor SGC	all	60.3	NA	NA	NA	293	52	1.31	0.751–2.28	0.341
Xu et al^30^.	2021	Major and minor SGC	ACC	56.4	NA	5.7 years	NA	26	29	1.113	NA	0.897
Yan et al^25^.	2024	Major SGC	all	38.6	NA	6.9 years	NA	4311	1116	1.04	0.92–1.19	0.519
Zhang et al^46^.	2023	Major SGC	SDC	55.1	NA	4.0 years	NA	71	71	1.408	0.809–2.45	0.226

PORT = postoperative radiotherapy; PORCT = postoperative radiochemotherapy; FU = follow-up; ACC = adenoid cystic carcinoma; SDC = salivary duct carcinoma; HR = hazard ratio, CI = confidence interval; NA = not available.

Only five of the included studies specified the chemotherapeutic agents used for PORCT, and each of these studies predominantly used platinum-containing regimens^26–30^. Six trials contained information on the applied radiation dose and five studies described the radiation techniques used^23,26–30^. In each of the studies, intensity-modulated radiotherapy (IMRT) was used in most cases, while a small percentage was treated with 2/3D-conformal radiotherapy (2/3D-CRT). Volume modulated arc therapy (VMAT) was used in one study^23^. An overview of the treatment modalities can be found in Table 2.

**Table 2:** Therapy modalities of the included studies.

Study	Chemotherapy, biological	Radiation dosage	Radiation technique
Amini et al^21^.	NA	NA	NA
Cheraghlou et al.^22^	NA	minimum 44 Gy	NA
Gordon et al^23^.	NA	minimum 60 Gy	IMRT, 2D/3DCRT
Kang et al^44^.	cisplatin, 5-fluorouracil, cyclophosphamide, carboplatin, docetaxel und methotrexate, cetuximab	66 Gy (60–70)	IMRT, VMAT, 2D/3DCRT
Onderdonk et al^27^.	5-FU + hydroxyurea + paclitaxel, 5-fluorouracil + hydroxyurea, cisplatin,	66 Gy RT: 50–75 Gy RCT: 43,2–70 Gy	IMRT, 3DCRT
Qiu et al^28^.	cisplatin, nedaplatin	RT: 68 Gy (60–74 Gy), RCT: 67 Gy (60–70 Gy)	IMRT
Tanvetyanon et al^29^.	cisplatin, cetuximab, carboplatin	NA	IMRT
Torabi et al^24^.	NA	NA	NA
Xu et al^30^.	cisplatin	62 Gy (46–72 Gy)	IMRT
Yan et al^25^.	NA	NA	NA
Zhang et al^46^.	NA	NA	NA

Gy = Gray; IMRT = intensity-modulated radiotherapy; 2/3DCRT = 2/3D-conformal radiotherapy; VMAT = volumetric modulated arc therapy; NA = not available.

### Meta-analysis on overall survival

**Fig. 2:**
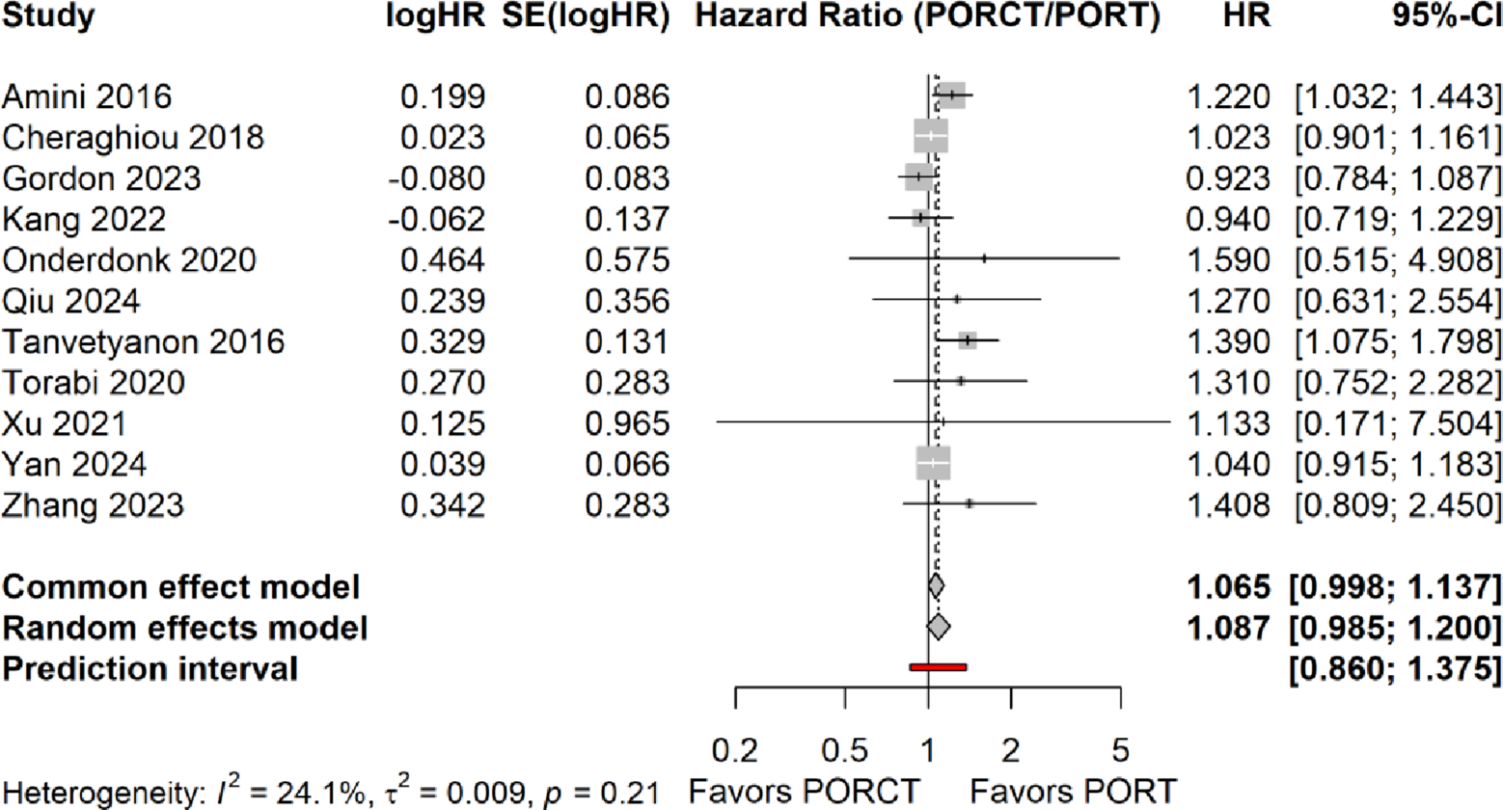
Forest plot for the meta-analysis of POCRT versus PORT with regard to the endpoint overall survival. HR = hazard ratio, SE: standard deviation of the effect size; CI: confidence interval, HK: Hartung-Knapp method. PORT: postoperative radiotherapy; PORCT: postoperative radiochemotherapy. Because of the low between study heterogeneity (I²=24.1%). Therefore, the common effect model (and not the random effect model) was referred for our primary analysis. Nevertheless, the data of the random effect model is also presented in the figure.

Based on a common effect model this meta-analysis revealed no statistically significant difference between PORT and PORCT (Fig. 2). The pooled HR was 1.065 with 95% CI (0.998; 1.137). When using alternatively the random effects model, the pooled HR was 1.087 with 95% CI (0.995; 1.120). So, both models produce very similar results. The 95% prediction interval ranged from 0.860 to 1.375, which indicates the estimated effect size for future studies, including a HR equal to 1.

### Between study heterogeneity

In terms of heterogeneity Cochran’s Q-test statistic equals 13.180, df = 10, *p* = 0.214. The corresponding I^2^ =24.1%. This indicates low heterogeneity. Likewise, the heterogeneity variance τ^2^ takes a value of 0.009 when a random effects model is applied. This also indicates low heterogeneity. As a result, a common effect model was applied for our primary analysis. For completeness the overall HR of the random effects model equals 1.087 with 95% CI (0.985; 1.200).

### Leave-one-out analysis

**Fig. 3:**
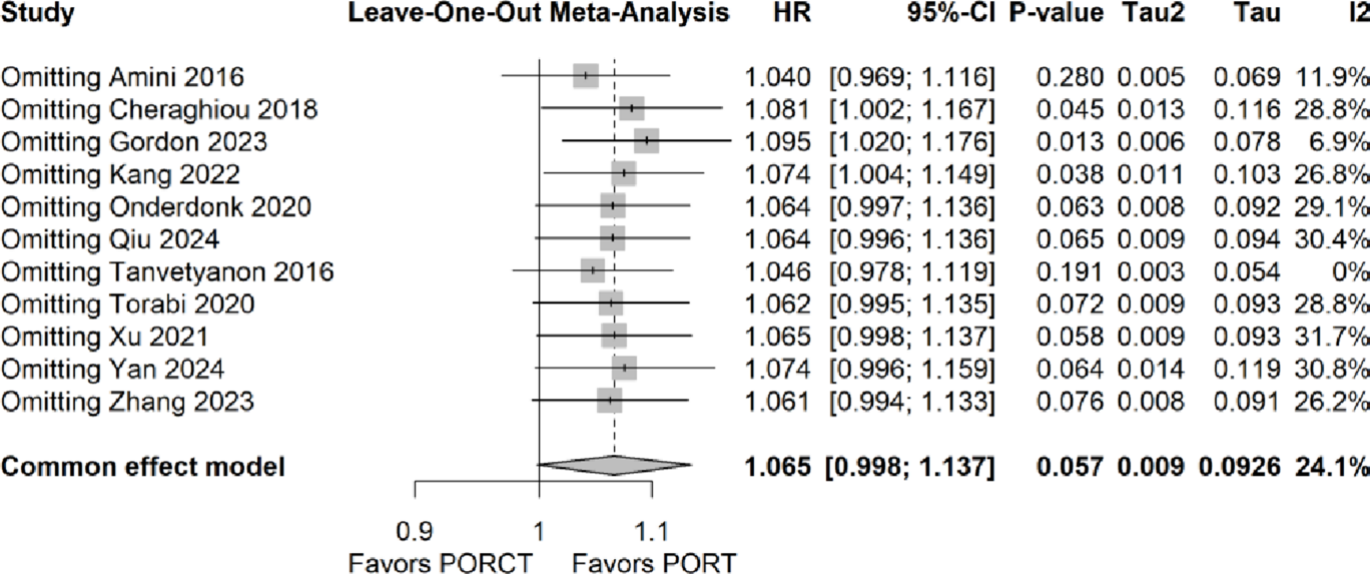
Leave-one out analyses. HR: hazard ratio; CI = confidence interval; I^2^: heterogeneity; tau: pooled effect size.

In the leave-one-out analysis the large studies by Amini et al. 2016, Cheraghiou et al. 2018, and Gordon et al. 2022 were identified as studies with a moderate influence on effect size and heterogeneity. Figure 3 shows the results of this analysis.

### Subgroup analysis and meta-regression

**Fig. 4:**
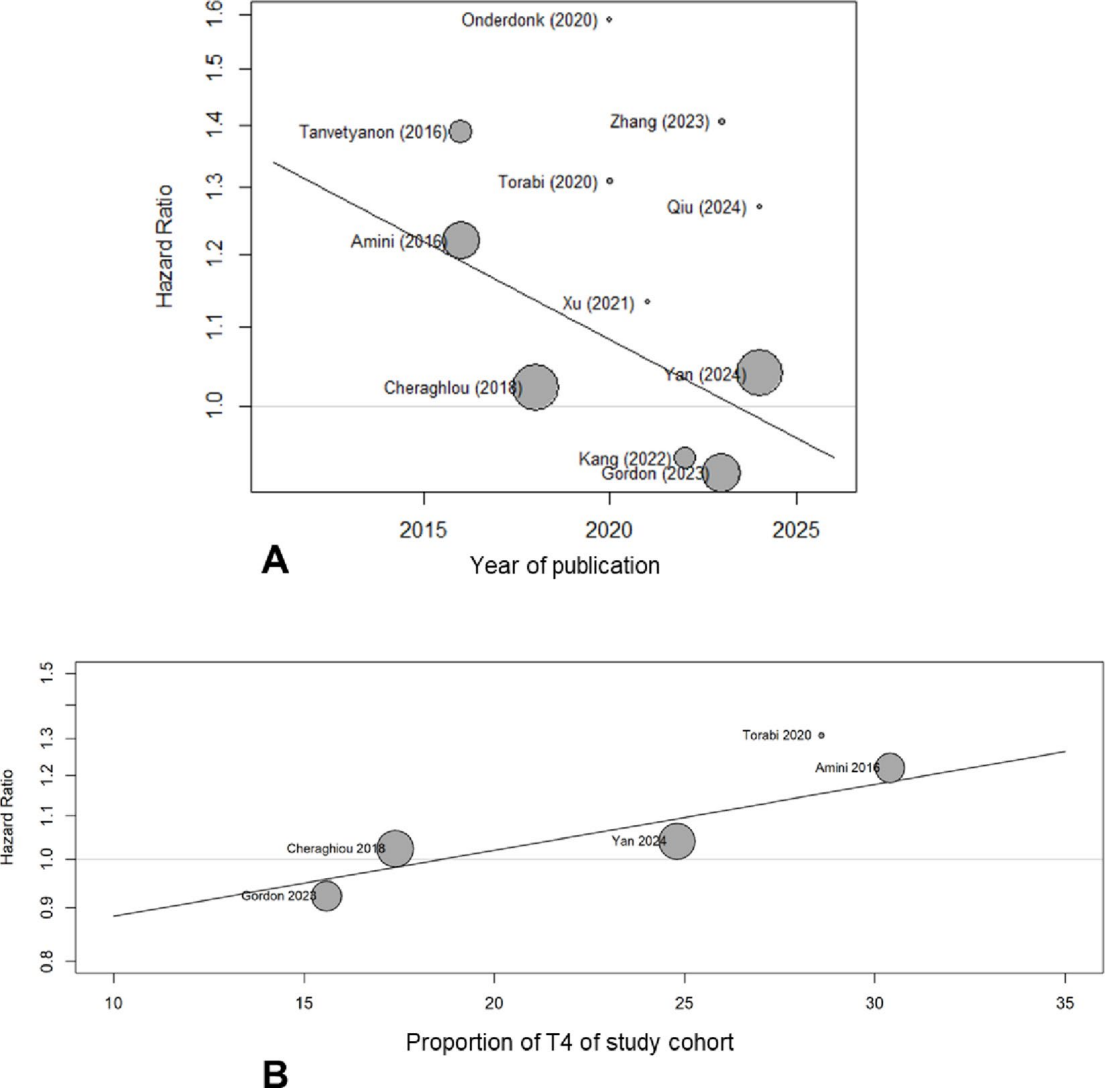
Bubble plots. **A**: for the meta-regression due to publication year. **B**: for the meta-regression due to proportion of patients with T4 salivary gland cancer out of all patients of the study sample.

Tumor and lymph node classification, histology and year of publication are possible factors influencing the probability of patient survival. Meta-regressions were used to quantify these potential effects (Table 3). Since five studies obtained their patient data from the same database, the difference between the NCDB subgroup and the remaining studies was analyzed for significance. The meta-regression for the year of publication showed no statistically significant result (*p* = 0.068). The pooled effects did not differ significantly (HR_NCDB_ = 1.05/HR_non-NCDB_ = 1.2; *p* = 0.150). The corresponding bubble plot is shown in Fig. 4A.

**Table 3:** Meta-regression analysis.

Item	Number of studies	*p*	Regression coefficient g	95% CI
Publication year	11	0.068	− 0.024	−0.048–0.0021
Proportion T3/4 of the total study cohort	10	0.350	0.003	−0.004–0.009
Proportion T4 of the total study cohort	6	0.039	0.0143	0.001–0.028
Ratio of T4 in PORCT/PORT	4	0.920	− 0.016	− 0.64–0.61
Ratio of N + in PORCT/PORT	6	0.099	− 0.059	− 0.13–0.017
Proportion of MEC of the total study cohort	10	0.950	− 0.0003	− 0.012–0.011

PORT = postoperative radiotherapy; PORCT = postoperative radiochemotherapy; MEC = mucoepidermoid carcinoma.

It is reasonable to assume that a higher tumor classification leads to shorter survival. Therefore, the proportion of tumors classified as T3/T4 and only as T4 in the total cohort, but also the ratio of tumors classified as T4 in the two intervention groups within the individual studies was examined in the meta-regression, provided that these or the data necessary for the calculation were given in the publications. A statistically significant covariate was only found for the percentage of T4 tumors in the overall cohort. The hazard ratio for a 10% change is given by HR = 1.154, with 95% CI (1.014; 1.314). Figure 4B shows the corresponding bubble plot. No significant effect on HR could be demonstrated for the proportions of high tumor grade and positive lymph node status in the intervention groups, although the corresponding patient data were only provided in four (T4) and six (N+) studies. For the sake of clarity, only the result for MEC is shown as an example for histology (cf. Table 3), which had the largest percentage share in most of the included studies. There was no significant effect for any histology.

### Publication bias

**Fig. 5:**
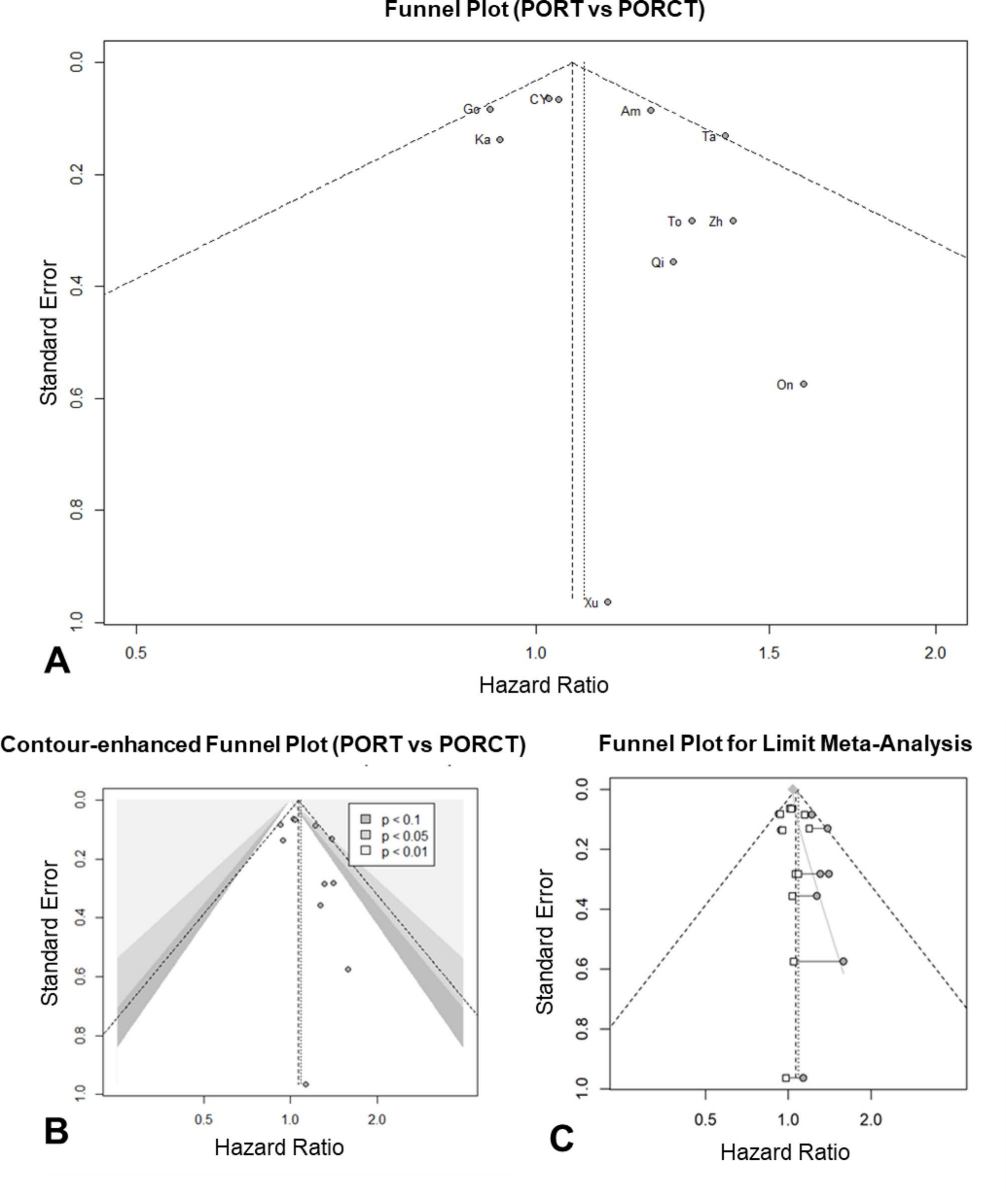
Forest plots. **A**: postoperative radiotherapy (PORT) versus postoperative radiochemotherapy (PORCT). **B**: Contour-enhanced Funnel plot. **C**: Funnel plot for limit meta-analysis. Am = Amini et al.^21^; C= Cheraghlou et al.^22^; Go= Gordon et al.^23^; Ka = Kang et al.^44^; On = Onderdonk et al.^27^; Qi = Qiu et al.^28^; Ta = Tanvetyanon et al.^29^; To = Torabi et al.^24^; Xu = Xu et al.^30^; Y = Yan et al.^25^; Zh = Zhang et al.^46^.

The funnel plot shown in Fig. 5A only showed a slight asymmetry in studies with a higher standard deviation. No publication was clearly outside the calculated ideal limits. To differentiate an asymmetry due to publication bias from an uneven distribution of studies caused by other factors, a contour funnel plot was then applied (Fig. 5B). Only two studies had significant effects^21,29^. A lack of publication of non-significant results could therefore not explain the low asymmetry. Egger’s regression test was carried out to objectify the funnel symmetry. This showed an intercept of 0.823 (CI −0.305–1.969, t = 1.433; *p* = 0.186). Thus, no significant asymmetry of the funnel plot could be detected. To validate this result, PET-PEESE and a limit meta-analysis according were then performed to calculate the true effect size after taking small-study effects into account (Fig. 5C). Here, too, there were no major differences in the pooled effect sizes. In the limit meta-analysis, the pooled hazard ratio based on a random effects model was HR = 1.040 95% CI (0.921; 1.174, *p* = 0.524).

### Systematic review on therapy toxicity

Only four included studies provided information on treatment side effects^27–30^. Even in these four studies the toxicity reporting is incomplete or inconsistent. Major deficits were a lack of treatment group stratification (toxicity after PORT vs. PORCT), and the absence of standardized toxicity grading like the CTCAE. Given the limited number of contributing studies and the substantial heterogeneity in reporting methods, the toxicity findings presented here should be considered exploratory only and are not suitable for comparative inference between PORT and PORCT.

Supplementary Table 3 summarizes the information on the side effects of the individual studies. The adverse effects observed in the four studies included nausea and vomiting, hematologic side effects such as anemia and neutropenia, as well as dehydration, dysphagia, infections, wound complications, pulmonary complications, renal insufficiency, skin toxicity, xerostomia, trismus, hearing loss, mucositis, and gastrointestinal toxicity.

When looking at the four studies individually, it becomes apparent that Xu et al. did not differentiate between patients who received PORCT and those who received PORT^30^. Severe toxicities (grade > 4) were not observed. Qiu et al. observed a significantly higher incidence of adverse events affecting the upper gastrointestinal tract and a higher incidence of hematotoxicity in the PORCT group, although grade 4 or higher RTOG toxicity was not reported for any of these organs^28^. Tanvetyanon et al. also found a higher incidence of treatment-related adverse events in the PORCT group in the multivariate analysis with an odds ratio (OR) of 6.79 (4.16–11.09). Nausea/vomiting, anemia, dehydration, infections, and wound complications occurred significantly more frequently in the PORCT group. There was a trend for more acute renal insufficiency and mucositis after PORCT, but statistical significance was not reached. In addition, a significantly more frequent extension of treatment duration > 3 months was found for patients treated with PORCT group, which was interpreted as an indication of more frequent toxicity-related treatment interruptions with a comparable median number of radiotherapy cycles^29^. In contrast, Onderdonk et al. found no significant differences in the treatment groups with regard to acute or late RTOG grade 3 or higher adverse events^27^. One grade 5 acute adverse event in the PORT group, but not in the PORCT group.

## Discussion

According to ASCO and ESMO/EURACAN Guidelines, PORT should be offered to all patients with adenoid cystic carcinoma, and for other salivary gland cancer types exhibiting high-grade differentiation, positive margins, perineural invasion, lymphovascular invasion, lymph node metastases, and pT3-4 tumors^31–33^. PORT should also be considered for patients with close margins or intermediate-grade tumors^32^. A recent meta-analysis confirmed that PORT demonstrates favorable long-term efficacy and safety in salivary gland cancer, particularly for patients with high-grade histology. The pooled overall survival rates at 3, 5, and 10 years were 84%, 75%, and 68% respectively^3^. Another meta-analysis focusing on adenoid cystic carcinoma found that PORT significantly improves local control, but failed to identify a statistically significant survival benefit at 5 and 10 years. The overall survival range in the included studies was 81–93% without PORT and 79–92% with PORT^2^. Hence, there is a need for treatment alternatives beyond PORT for salivary gland cancer, at least for patients wirh high-risk criteria.

In this meta-analysis and systematic review, pooled data from the included studies did not show improved OS with PORCT compared to PORT as adjuvant treatment after surgery. Due to the retrospective design of the included studies, results must be interpreted with caution. It cannot be ruled out that some patients with poorer risk profiles received PORCT rather than PORT, which biases prognosis from the outset. Most included studies pooled patients from major and minor salivary glands and encompassed a wide variety of histological subtypes. There is only limited evidence that patients with adenoid cystic carcinoma do not benefit from PORCT compared to PORT^28,30^. The included studies did not exclusively include high-risk patients (T3/4, N+, small margins, extracapsular spread). Therefore, future analyses of better-defined patient subsets may be valuable. Large randomized controlled trials for salivary gland cancer subsets are unlikely, hence, cancer registry-based studies using data drawn from the National Cancer Database (NCDB) or the Surveillance, Epidemiology, and End Results (SEER) program are particularly valuable^21–24,29^. Finally, the present meta-analysis focused solely on OS. Cancer-specific survival was not analyzed, likely due to insufficient information. Consequently, the causes of death in this meta-analysis remain unknown. A major problem of salivary gland cancer is distant metastasis as a driver for tumor recurrence^34^. PORCT does not appear to reduce the risk of late distant metastasis^35^.

Even if PORCT were be more effective than PORT in a subset of patients, it is important to note that PORT alone already leads to CTCAE grade 3 toxicities of approximately 31% of the cases, and to grade 1/2 or grade 3 toxicities in roughly 83% and 7%, of the cases, respectively^3,36^. Although, a meta-analysis to compare toxicity between PORT and PORCT was initially planned, the available data were insufficient. The systematic review suggested as expected that the risk of acute toxicity is higher after PORCT compared to PORT. Toxicity assessment remains critical when weighing the benefits of PORT against PORCT. Even if PORCT proves beneficial in terms of survival for a subgroup in the ongoing RTOG-1008 study (ClinicalTrials.gov identifier NCT01220583; more details, see below), this benefit must to be balanced against the potential for increased toxicity. The RTOG-1008 protocol includes acute toxicity comparison between PORT and PORCT.

Salivary gland cancer survivors are underrepresented in long-term head and neck cancer survivorship studies. Swallowing and eating problems appear more common in these patients compared to other head and neck cancer survivors^37^. Survivors who received chemotherapy have an elevated risk of long-term sequelae, significantly impacting quality of life^37,38^.

Several limitations of this study have to be acknowledged. All included studies were retrospective, posing a risk of selection bias. Patients receiving PORCT are generally more likely to have adverse baseline prognostic factors (e.g., higher T classification, higher N classification, other high-risk pathological features). Some (not all) of these factors could be studied by the meta-regression. T4 had a significantly higher probability to be found in the PORCT than in the PORT group and N+ showed a trend to be more frequent in the PORCT group. It has to be emphasized that only four of the selected eleven studies provided sufficient data to compare the proportions of T4 tumors in the PORCT group versus the PORT group. The small number of studies likely contributed to the fact that the corresponding meta-regression did not show statistical significance. Furthermore, in all studies included in the meta-regression, the percentage of T4 and N+ tumors was higher in the PORCT group than in the PORT group. The imbalance in the T and N classification between the PORCT and PORT group posed an important bias, potentially driving the results toward no observable OS effect between both groups. Consequently, the absence of a statistically significant survival benefit for PORCT in this meta-analysis must be interpreted with caution and may, at least in part, reflect this underlying selection bias rather than a true absence of a treatment effect. In optimally balanced groups, one would have expected that, on the one hand, a higher T-classification result in a lower OS, and on the other hand, that PORCT might be beneficial for high-risk groups.

The present meta-analysis exclusively focused on OS. Other clinically relevant endpoints such as disease-free survival, locoregional control, and distant metastasis-free survival could not be considered due to insufficient pooled data. This is another limitation, as these endpoints are as important as OS for the evaluation of treatment strategies for salivary gland cancer. The theoretical background of PORCT instead of PORT is not solely to improve OS in high-risk scenarios, but also to improve locoregional control and, especially for adenoid cystic carcinoma, potentially to reduce the risk of distant metastasis.

Heterogeneity in histological subtypes and anatomical locations posed additional challenges. Since there are no clear standards for this in salivary gland cancer, the radiotherapy protocols and chemotherapy regimens can also vary considerably. Pooling data without subgrouping was the only feasible approach, as several studies lacked consistent subgroup information and some subgroups were too small for analysis. In particular, a consequence of the pooling wa that we cannot rule out the possibility that treatment effects may differ across specific histological subgroups and subsites.

The ongoing RTOG-1008 phase II/III trial is investigating cisplatin-based PORCT versus PORT in resected high-risk salivary gland carcinoma with a 4-year follow-up (ClinicalTrials.gov identifier NCT01220583). This trial may clarify, whether patients with a higher T or N classification benefit from PORCT. Recruitment was started in 2010 and completed in March 2021, with first results expected in 2026. No other prospective trials comparing PORCT and PORT are currently ongoing.

If classical chemotherapy offers no additive value, what are the alternatives? Immunotherapy, while now standard of care in the recurrent/metastatic head and neck squamous cell carcinoma, has been disappointing for salivary gland cancer^39,40^. There are currently no prospective studies on adjuvant systemic therapy in combination with radiotherapy or instead of radiotherapy. Retrospective data suggest a survival benefit from adjuvant trastuzumab after surgery and PORT in Her2/neu expression DAKO3 + tumors^41^. Also according to a retrospective analysis, androgen deprivation therapy with bicalutamide or with an LH-RH agonist (goserelin, triptorelin) as monotherapy or in combination in stage IV androgen receptor-positive salivary duct carcinoma given as adjuvant therapy after PORT over 1–5 years could provide an advantage over radiotherapy alone^42^. However, there is currently a lack of evidence for the benefit shown in prospective clinical trials^43^. Therefore, one ongoing phase II study is on interest. This phase II trial is on adjuvant combination therapy with ado-trastuzumab emtansine (T-DM1) in HER2-positive salivary gland carcinomas during and after postoperative radiotherapy. It started in 2020 and is still recruiting (ClinicalTrials.gov Identifier NCT04620187). Collectively, these examples illustrate the slow progress in improving treatment for salivary gland cancer.

## Conclusions

PORT after in operated high-risk salivary gland cancer patients is the standard of care. However, the overall survival of many of these patients remains unsatisfactory. So far, there is no clear evidence of the benefits of PORCT after surgery for salivary gland cancer. This meta-analysis was performed to determine whether PORCT leads to better prognosis than PORT in patients undergoing primary surgery for salivary gland cancer.

A total of 11 retrospective clinical studies including 26,612 adult patients were analyzed. The meta-analysis could not demonstrate a benefit of PORCT compared to PORT with regard to overall survival. To avoid overinterpretation of the non-significant findings, it should be emphasized that there was substantial clinical heterogeneity across the included studies in terms of histology, primary tumor site (major vs. minor salivary glands), stage distribution, and treatment protocols (including chemotherapy regimens and radiotherapy techniques). The retrospective design of the included studies and this heterogeneity resulted in a high degree of uncertainty about the treatment effects. Treatment effects may differ across specific subgroups. More robust data from high-quality cohort studies or randomized controlled studies are urgently needed. Therefore, the results of the RTOG-1008 phase II/III trial will be very important. This trial will answer the question whether patients with operated high-risk salivary gland carcinoma benefit from PORCT with cisplatin compared with PORT. Beyond this, there is an urgent need for alternatives to classical chemotherapy as adjuvant treatment in patients with operated high-risk salivary gland cancer.

## Supplementary Information

Below is the link to the electronic supplementary material.

Supplementary Material 1

## Data Availability

Data is provided within the manuscript. All authors had full access to all of the data in the study. O.G.-L. and P.S. take responsibility for the integrity of the data and the accuracy of the data analysis. O.G.-L. should be contacted if someone wants to request the data from this study.

## Declarations

### Generative AI and AI-assisted technologies in the writing process

During the preparation of this work the authors did not use such a technology.
