# Supplementary material for: Postoperative radiotherapy versus postoperative radiochemotherapy after surgery of salivary gland cancer: a systematic review and meta-analysis
Source: Sci Rep. 2026 May 6;16:14426. doi: 10.1038/s41598-026-52018-4 (PMC13149990; doi:10.1038/s41598-026-52018-4)
Supplement: Supplementary file 1 — Supplementary Material 1 [file 41598_2026_52018_MOESM1_ESM.docx]

**Supplementary Material**

**Postoperative radiotherapy versus postoperative radiochemotherapy after surgery of salivary gland cancer: a systematic review and meta-analysis**

Anja Wilhelmy, Peter Schlattmann, Orlando Guntinas-Lichius^3^

**Supplementary Table 1**. Reasons for exclusion of studies during the full-text analysis.

**Supplementary Table 2.** Assessment of the included studies with the Newcastle–Ottawa Scale (NOS).

**Supplementary Table 3.** Reported toxicity in the included studies.

**Supplementary Table 1**

| **Supplementary Table 1**. Reasons for exclusion of studies during the full-text analysis. | | |
| --- | --- | --- |
| **Reason** | **Number of studies** |  |
| Study was not available in German or English | 5 |  |
| Review, case report or a single case study | 11 |  |
| Including patients with recurrent disease or with distant metastasis (M+) | 70 |  |
| Other primary cancer | 5 |  |
| No separation between PORT and PORCT | 30 |  |
| No overall survival data in relation to PORT and PORCT | 41 |  |
| Not reporting hazard ratios | 27 |  |
| Sum of excluded studies | 204 |  |

PORT = postoperative radiotherapy; PORCT = postoperative radiochemotherapy.

**Supplementary Table 2**

| **Supplementary Table 2. Assessment of the included studies with the Newcastle–Ottawa Scale (NOS).** | | | | | | | | | | | | | | | |
| --- | --- | --- | --- | --- | --- | --- | --- | --- | --- | --- | --- | --- | --- | --- | --- |
| **References*** | **Clareness of stated aim (0–2)** | **Sample selection** | | | | | **Comparability** | | | **Outcome** | | **NOS score (0–16)** | **Quality** | |  |
|  |  | Sample representativeness (0–2) | Sample size (0–2) | Non-respondents (0–2) | Exposure Assessment (0–2) | Control of confounding factors (0–1) | | Comparability of participants (0–1) | Assessment of the outcome (0–2) | | Statistical tests (0–2) |  |  |  |  |
| Amini et al. [^21^](#_ENREF_21) | 2 | 2 | 2 | 0 | 2 | 1 | | 1 | 2 | | 2 | 14 | high |  |  |
| Cheraghlou et al. [^22^](#_ENREF_22) | 2 | 2 | 2 | 0 | 2 | 1 | | 1 | 2 | | 2 | 14 | high |  |  |
| Gordon et al. [^23^](#_ENREF_23) | 1 | 2 | 2 | 0 | 2 | 1 | | 1 | 2 | | 2 | 13 | high |  |  |
| Kang et al. [^44^](#_ENREF_44) | 1 | 1 | 2 | 0 | 2 | 1 | | 1 | 2 | | 2 | 12 | moderate |  |  |
| Onderdonk et al. [^27^](#_ENREF_27) | 1 | 1 | 1 | 0 | 2 | 1 | | 1 | 2 | | 2 | 11 | moderate |  |  |
| Qiu et al. [^28^](#_ENREF_28) | 1 | 1 | 1 | 0 | 2 | 1 | | 1 | 2 | | 2 | 11 | moderate |  |  |
| Tanvetyanon et al. [^29^](#_ENREF_29) | 1 | 2 | 2 | 0 | 2 | 1 | | 1 | 2 | | 2 | 13 | high |  |  |
| Torabi et al. [^24^](#_ENREF_24) | 1 | 2 | 2 | 0 | 2 | 1 | | 1 | 2 | | 2 | 13 | high |  |  |
| Xu et al. [^30^](#_ENREF_30) | 1 | 1 | 0 | 0 | 1 | 1 | | 1 | 2 | | 2 | 9 | moderate |  |  |
| Yan et al. [^25^](#_ENREF_25) | 1 | 2 | 2 | 0 | 2 | 1 | | 1 | 2 | | 2 | 13 | high |  |  |
| Zhang et al. [^46^](#_ENREF_46) | 1 | 2 | 1 | 0 | 2 | 1 | | 1 | 2 | | 2 | 12 | moderate |  |  |

*Reference numbers related to the Reference list in the main manuscript.

**Supplementary Table 3**

| **Supplementary Table 3**. Reported toxicity in the included studies. | | | | |
| --- | --- | --- | --- | --- |
| **Study*** | **PORT**  **(n)** | **PORCT (n)** | **Classification** | **Side effects** |
| Onderdonk et al. [^27^](#_ENREF_27) | 50 | 58 | RTOG | - No significant difference in acute toxicity grade ≥3 (PORCT 36.2 %, PORT 36.0 %, p = 0.98). - No significant difference in late toxicity grade ≥3 (PORCT 6.9 %, PORT 6.0 %, p = 0.85). - One grade 5 acute adverse event in the PORT group. |
| Qiu et al. [^28^](#_ENREF_28) | 147 | 59 | RTOG | - Significantly more frequent acute upper gastrointestinal adverse events and hematotoxicity in the PORCT group (gastrointestinal grade 1 PORCT 64.4 %, PORT 34.7 %, p < 0.001; hematologic grade 0-1 PORCT 74.6 %, PORT 96.6 %, grade 2-3 PORCT 25.4 %, PORT 3.4 %, p < 0.001) |
| Tanvetyanon et al. [^29^](#_ENREF_29) | 641 | 100 | NA | - Significantly more frequent side effects in PORCT group (PORCT 72 %, PORT 27.3 %, p ≤0.001) and longer duration of therapy as an indication for more frequent interruption of therapy (PORCT 29.3 %, PORT 20.2 %, p = 0.039). - In PORCT group significantly more frequent nausea/vomiting (PORCT 33 %, PORT 6.2 %, p≤0.001), anemia (PORCT 29 %, PORT 13.9 %, p = 0.001), dehydration (PORCT 18 %, PORT 3, 7 %, p≤0.001), infections or wound complications (PORCT 15 %, PORT 5.5 %, p = 0.001) and acute renal insufficiency or mucositis (PORCT 12 %, PORT 4.7 %, p = 0.100) |
| Xu et al. ^30^ | 26 | 29 | CTCAE v4.0 | - No data on side effects depending on the adjuvant treatment modality, no grade ≥4 toxicity in the overall cohort. |

*Reference numbers related to the Reference list in the main manuscript.; PORT = postoperative radiotherapy; PORCT = postoperative radiochemotherapy; RTOG = radiation Therapy Oncology Group; CTCAE = Common Terminology Criteria for Adverse Events; NA = not available.
